# Generation of bioinspired structural colors via two-photon polymerization

**DOI:** 10.1038/s41598-017-17914-w

**Published:** 2017-12-15

**Authors:** Gordon Zyla, Alexander Kovalev, Markus Grafen, Evgeny L. Gurevich, Cemal Esen, Andreas Ostendorf, Stanislav Gorb

**Affiliations:** 1 Applied Laser Technologies, Ruhr-Universität Bochum, Universitätsstraße 150, 44801 Bochum, Germany; 2 Functional Morphology and Biomechanics, Christian-Albrechts-Universität zu Kiel, Am Botanischen Garten 9, 24098 Kiel, Germany

## Abstract

Colors of crystals, pigments, metals, salt solutions and bioluminescence occur in nature due to the optical properties of electrons in atoms and molecules. However, colors can also result from interference effects on nanostructures. In contrast to artificial coloration, which are caused by well-defined regular structures, the structural colors of living organisms are often more intense and almost angle-independent. In this paper, we report the successful manufacturing of a lamellar nanostructure that mimics the ridge shape of the Morpho butterfly using a 3d-direct laser writing technique. The viewing angle dependency of the color was analyzed via a spectrometer and the structure was visualized using a scanning electron microscope. The generated nano- and micro-structures and their optical properties were comparable to those observed in the Morpho butterfly.

## Introduction

The optical properties of biological surfaces are important for biological organisms. Light scattering contributes to various biological functions, such as camouflage and mimicry^1^, and in some cases, the survival of a living organism is based on its coloration. Other basic functions of color in living organisms include display functions for communication with a mate during courtship^2^ or for sending warning signals to rivals^3^.

The most common colors in biological systems are those from pigments. Biological surface structure modification is an alternative color formation mechanism. In biological systems, structural coloration is caused by layered structures, layers of particles/fibrils/sheets, 3d photonic crystals or surface micro- and nano-structures, and it can also be supported by pigments^4^. The disorder in the spatial distribution of the structures and their periodicity have important roles in the specific optical properties of the coloration^5^.

Color formation induced by multilayer interference is typically based on highly ordered structures. Among the many examples of this type of structural coloration^4,5^, one of the most famous is that of the *Morpho* ^6^ butterfly from South America, especially *M. didius* or *M. rhetenor*. While most organisms with colors based on micro- and nano-structures show a rainbow-like, shimmering iridescence, e.g., the feathers of birds^4^, the blue color of *M. rhetenor* is intense and can be observed over a wide range of viewing angles^7^. The *Morpho* scales are structured and ordered on a few hierarchical levels, but the scales contain simple defects and/or random variations in the shapes of the structures on each level. Overall, this leads to interference, diffraction and scattering^8^.

In general, the artificial generation of coloration encountered in nature, which does not rely on toxic dyes or synthetic pigments, is highly interesting for potential technical applications, e.g., textile apparel^9^, selective gas^10^ and high-speed infrared imaging devices^11^. Yang *et al*.^12^ presented angle-insensitive, reflective color filters in the entire visible range based on Fabry-Perot cavities using different nickel layers as a broadband absorber which were fabricated with electron-beam vapor deposition. Kumar *et al*.^13^ produced coloration through plasmonic structures using nanoimprint technology and metal vapor deposition. These coloration^12,13^, showed interesting optical properties but the generation is based on multiple fabrications steps, requires multiple materials and the coloration does not result from biomimetic structures. The interdisciplinary linkage between engineering and biology can be highly interesting and may pave the way for enhanced understanding of biological photonic systems on one side and the development of technical photonic systems with novel optical properties on the other side. Besides, biological micro- and nano-structured surfaces are normally multifunctional. The surfaces may enhance the transparency of a material by reducing its reflection^14^, wear resistance^15^ or superhydrophobicity^16,17^, These properties are also important for engineered materials with a broad range of real world applications.

There are numerous publications on the origin of the colors in biological photonic systems and some of these systems have been successfully mimicked. However, the large-scale production of bioinspired photonic structures is still a challenge. Previous authors have reported on color formation mechanisms and on the use of spectral shifts in the structural colors of multilayered systems containing materials with different refractive indices to sense temperature^18^ or mechanical^19,20^, and chemical properties^21,22^. Furthermore, mimics of the *Morpho* color formation have been reported in numerous publications using techniques such as multilayer deposition of TiO_2_/SiO_2_^23^ or SiO_2_/SiO_4_^24^, focused-ion-beam chemical-vapor-deposition (FIB-CVD)^25^ and synthese of 3d nanostructures using metal oxides^26–28^, or polydimethylsiloxane^29^.

Color formation based only on the fabrication of micro- and nano-structures with lithographic methods offers many advantages not available in coated multilayer systems because it avoids multiple complex fabrication steps. In this field, electron beam lithography can replicate the *Morpho* lamellar structure and its blue coloration^30–32^. However, the manufacturing procedure for electron beam lithography is complex due to the usage of individual masks and a vacuum for the structuring. Whereas, two-photon polymerization (2PP) is a more flexible lithographic fabrication process that can generate arbitrary 3d structures. Unlike with other lithography methods, with 2PP, a resolution of less than 100 nm can be easily achieved without the requirement of etching, masks and vacuum. Thus far, the smallest feature produced using the 2PP technique was 26 nm^33^. The polymerization of the photoresists is based on the quasi-simultaneous absorption of two photons^34,35^. The process triggers a chemical reaction in a photosensitive molecule, the photoinitiator. Both photons are absorbed within a time interval of several femtoseconds. This allows the polymerization reaction to be induced near the center of the focal spot and reduces the influence of the light scattered by the structure that is produced. Different photosensitive materials with various properties can be used for the process. The resulting 3d structures with arbitrary shapes have previously been studied in a broad range of research fields^36–38^, In this context, 2PP allows different color formations to be studied via the fabrication of transmission phase gratings as color filters^39^. Complex, tarantula-inspired structures allow investigations of the non-iridescent properties of structural coloration^40^. Furthermore, 3d woodpile photonic crystals have been used to generate different colors by varying the lattice periods^41^.

In the present paper, we demonstrate a concept and fabrication method to mimic the structural coloration of the *Morpho* butterfly using 2PP. A scanning electron microscope (SEM) was used for the characterization of the photonic 2PP-nanostructures. The identification of the influence of the structures on the artificial color formation were performed using angle-resolved spectroscopy.

## Results

### Concept for the biomimetic fabrication of *Morpho* lamellas using 2PP

**Figure 1:**
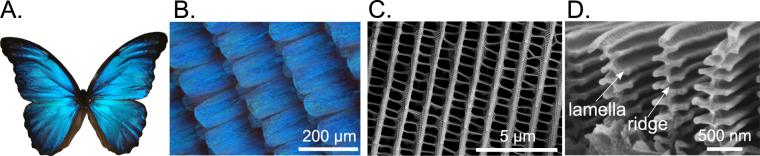
Structural coloration and wing composition of the *M. didius*. The blue coloration caused by the surface micro-/nano-structures is shown for a specimen of a *M. didius* in (**A**). The blue colored scales of the *M. didius* are presented in (**B**). A scanning electron microscope (SEM) image in (**C**) illustrates the arrangement of the *M. didius* ridges on a single wing scale. The cross section of the *Morpho* ridges and their Christmas tree shape contains alternating layers of air and natural material (cuticula) in the form of lamellas, as shown by the SEM image^30^ (**D**).

Although biological structural color systems are described as highly ordered, they contain also disorder. To develop a flexible fabrication method to analyze color formation based on hierarchical structures with disorder, a proper integration of the micro- and nano-structures must be achieved. In contrast to other lithography methods, 2PP offers this flexibility due to the maskless fabrication of arbitrary structure shapes and its simple production process. To understand the biological structural color mechanism, mimicking the *Morpho* butterflies’ color formation and its observation angle independence is of great interest. However, the structural composition of the *Morpho* scales is highly complex (see Fig. 1A–C). The wing surface of *M. didius* is covered in a high number of scales, and the scales contain periodic, identical, multilayered ridges. The shape of a single ridge in the cross section, as illustrated in Fig. 1D, is similar to the lamellar structure of a Christmas tree. The height of the ridges differ between 1.2 μm and 1.6 μm^8^ based on the genus of the *Morpho* and they consist of varying multiple layers of cuticula^42,43^.

**Figure 2:**
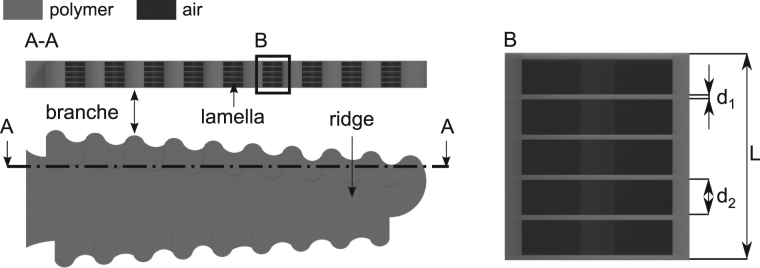
Computer-aided design of a single artificial photonic structure. The shape of the photonic structure on micron scale mimicking the artificial branches and ridge is shown in the top view. The selection drawing (**A-A**) illustrates the periodicity in the cross section of the microstructure. The periodicity consists of thin polymer conjunctions between each single branch, which mimic the *Morpho* lamellas. The artificial polymer lamellas are separated from each other through air cavities. The enlarged section (**B**) demonstrates the composition of one periodicity in detail. The thickness of one thin polymer film is defined with the value *d*_1_. The size of one air is referenced with the value *d*_2_. The parameter *L* denotes the total length of the periodicity.

Although 2PP possesses a high lateral resolution in the sub-100 nm range, an accurate replicate of a single *Morpho* wing scale is difficult to fabricate due to the lower axial resolution of 2PP, the small size of the features, and the high aspect ratio and high level of detail in the *Morpho* lamella. Therefore, the design was adapted to mimic the blue coloration of the *Morpho* butterfly using the 2PP technique for the first time. The resulting model is shown in Fig. 2. The color formation is generated by the same fabricated microstructures, which are assembled in a defined array. The microstructures possess a hierarchical construction, which contains artificial ridges, branches, nanoscale polymer lamellas and air cavities. The cross section of the bioinspired microstructure is comparable to the shape of the *Morpho* Christmas tree. The thickness of the polymer lamellas and air cavities inside the microstructure were designed based on the maximum constructive interference condition of a multilayer stack with two different materials *λ*_*max*_=2(*n*_1_ *d*_1_ + *n*_2_ *d*_2_)^44^ in the case of perpendicular incident light (see. Fig 2). While *n*_1_ and *d*_1_ denote the refractive index and layer thickness of the polymer lamellas, respectively, the index 2 belongs to the parameters of air. The basic design is illustrated in Fig. 2, in which *L* is the total length of the periodic structure. Dimensions of an artificial structure for a blue *Morpho*-like coloration^6–8^, can be chosen as follows. Artificial lamellas with *n*_1_ = 1.51 and *d*_1_ = 50 nm and air cavities with *n*_2_ = 1 and *d*_2_ = 150 nm. These estimations agree with the microscope studies of the *Morpho* butterfly^42,43^.

**Figure 3:**
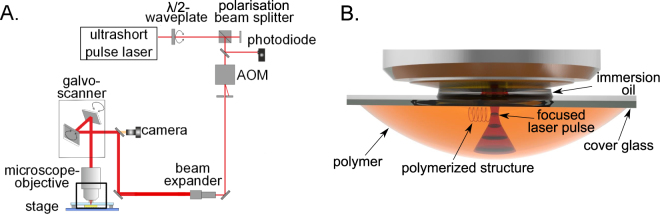
Experimental 2PP set-up and detailed view of the polymerization inside a photosensitve material. The 2PP setup is illustrated schematically with an ultrashort pulse laser and a microscope objective (NA = 1.4) in (**A**). The principal of the 2PP manufacturing process inside a volume of polymer is demonstrated in (**B**).

The principal methods and tools for the generation of structural colors using hierarchically combined micro- and nano-structures, including the experimental setup, and a detailed view of the polymerization process are shown in Fig. 3. A microstructure array of 250 μm× 250 μm was fabricated with regard to a low production time and a representative analysis option for the structural color formation. The photonic structures were generated layer by layer. Each 2PP ridge layer (see Fig. 2) served as a spacer between the 2PP branches. Furthermore, the short lateral distances between the branches inside a single layer enhances curing the weakly polymerized regions via the diffusion of radicals^45^ in the nanometer range. Thus, a thin polymer junction can be produced in this section. In this way, artificial lamellas and interlayered air cavities in the nanometer range can be built.

### Characterization of bioinspired photonic structures

**Figure 4:**
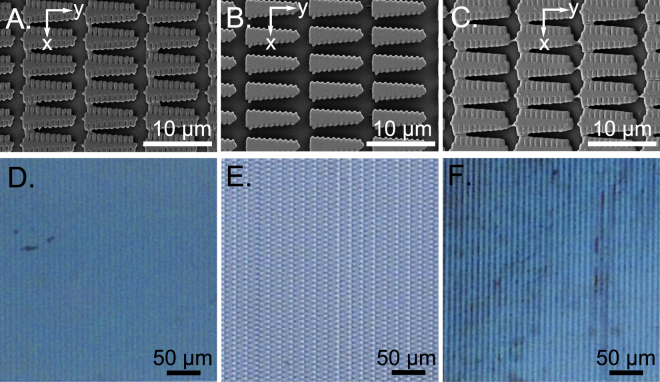
Artificial blue coloration fabricated with photonic structures using 2PP. A section view of the polymerized array of three different laterally scaled photonic structures from the actual structure geometry (see Fig. 2) are shown in the SEM images (**A**)–(**C**). In comparison to sample (**A**), the variation in the lateral distances between each microstructure is illustrated in the SEM images for samples (**B**) and (**C**). The microscope images in (**D**)–(**F**) demonstrate the color formation results from those arrays.

Uniform lamellar structures were generated in a thin polymer film. The film thickness was completely exposed to compensate for the roughness of the glass substrate. Additionally, further interference effects or diffraction via irregularities in the microstructure heights and differences in the laser radiation exposure during the production process can be avoided. A total polymer film thickness of 1 μm was used to generate the photonic structures for the structural coloration. The film thickness was verified using a white light interferometer after the specimen was cured for 24 h using UV light. The scanning electron microscope (SEM) images give an overview of the similarities among the individual structures inside the relevant area (see Fig. 4A–C). The photonic structures of all the samples were fabricated with the same number of layers. An analysis of the influence of the microstructure on the resulting color was performed using three different scaling values for the lateral microstructure shape and a difference in the microstructures’ density in a 250 μm × 250 μm array. The density was controlled by the variation in the lateral *x*,*y*-distances between each microstructure (see Fig. 4A–C). A microscope with a standard halogen lamp was used for the first estimation of the light reflection from the samples. The microstructure top layer in sample A (see Fig. 4A) illustrates a nearly perfect polymerization of the actual lateral structure shape (compare Fig. 2), but the top layers of sample B and C (see Fig. 4B,C) feature higher polymerization degrees due to the lateral size differences in the scaled shape of the microstructures. However, a thin polymerization junction between single branches can be identified for all samples. This indicates the potential to generate nanometer lamellas and a hierarchical design inside the microstructures. Nevertheless, a variation in the actual microstructure shape did not affect the light reflection properties. All arrays ensured blue color formation (see Fig. 4D–F). Furthermore, the blue color hue was independent of the density of microstructures inside the array, as illustrated by the microscope images in Fig. 4. A decrease of the microstructure density (see. Fig. 4E) only affects a lower color intensity compared to the more densely packed samples as shown in Fig. 4D and F.

**Figure 5:**
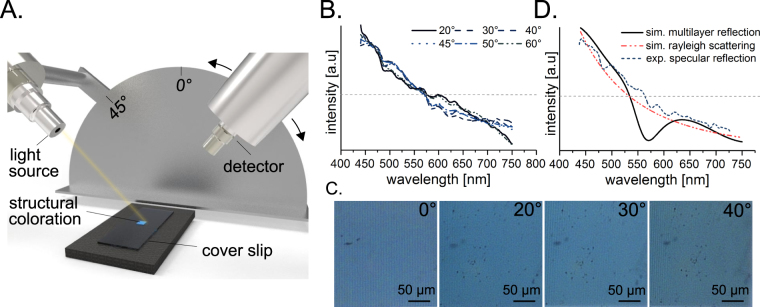
Light scattering properties of photonic structures. The principal setup for spectral measurement of the coloration depending on the observer’s angle is illustrated in (**A**). A sample was illuminated at 45° and reflection spectra were measured at different angles. (**B**) The normalized to their area reflection spectra are shown at different observation angles. Upright light microscopy images (**C**) at different sample tilt angles were obtained using a microscope color camera. The simulation results of reflection spectra for the artificial photonic structure using two different approaches is illustrated in (**D**). A specular reflection for multilayer system consisting of 5 layers of the polymer separated by air layers is shown by solid line. Rayleigh scattering on multiple subwavelength centers is shown by dot/dash-line. Furthermore, the dash-line represents the specular reflection of the real photonic structure.

To study the color formation as a function of the viewing angle, the spectrum of sample A (see Fig. 4A,D) was measured using the setup illustrated in Fig. 5A. The spectral results of variable viewing angle from 20° to 60° can be seen in Fig. 5B. We expected to observe a set of characteristic diffraction peaks at each position that were based on the observation angle or a characteristic spectrum corresponding to the Rayleigh light scattering with an intensity of ~*λ* ^−4^ and low angular dependency. Surprisingly, the measured spectra did not resemble either of the expected results. In contrast, the spectrum was independent of the observation angle, which is similar to the property demonstrated by the biological butterfly template^6–8^. Furthermore, images of the sample A were taken under a microscope with a camera at 0°, 20°, 30°, 40° observation angles (see Fig. 5C) to confirm the spectral data. The sample was homogeneously illuminated. The blue coloration was independent of the observation angle.

In order to determinate the color’s origin, the artificial structures have to be simulated numerically. Precise calculation of light scattering on 3d nanoscale gratings requires numerical solution of Maxwell’s equations and is rather complex. Here we evaluate different effects influencing the light reflection on our fabricated photonic structures by the combination of two models of light reflection (see Fig. 5D). An ideal periodic structure would act as a bragg mirror and the reflection will be angle-dependent and described by the maximum constructive interference condition^44^. A set of random Rayleigh scattering centers would scatter the light almost angle-independently according to *I* ∝ *I*_0_ *d* ^6^(*R* ^2^ *λ* ^4^)^−1^(1 + *cos* ^2^θ)^46^, where *θ* is the scattering angle, *d* is the size of the scattering center, *R* is the distance and *λ* is the wavelength. We suppose that the mainly part of the incident light intensity is reflected on the periodic structures. However, there is also a scattering of the incident light on the random Rayleigh scattering centers. Thus, the resulting field is a superposition of these two processes. The calculations of these two components of the scattered field can be analyzed as follows. Taking into account that the incident angle equals 45°, and considering the structure as a standard diffraction grating the specular reflection at 45° should be observed. The first order reflection should appear for observation angles around to the surface normal and demonstrate the gradual color change from blue to red. The second order should be observed in the backward direction. These properties were not observed for the structures in the real experiments. The next interpretation of the optical properties of the artificial photonic structures is the interference in thin films. The reflection angle should be equal to the incident angle, but because of the interference the specular reflection should be colored. Assuming the structures are monolithic and are 1 μm thick, the fourth and fifth order maxima of constructive interference should be in green and in red. Considering the presence of gaps between individual printed layers on the edges of the structures, and assuming that the thickness of both gaps and polymer layers is equal, the spectrum of specular reflection could be calculated (see solid line in Fig. 5D). Finally, the reflection less dependent on observation angle could be caused by Rayleigh scattering. In this case the reflection intensity is inversely proportional to the forth degree of wavelength.

**Figure 6:**
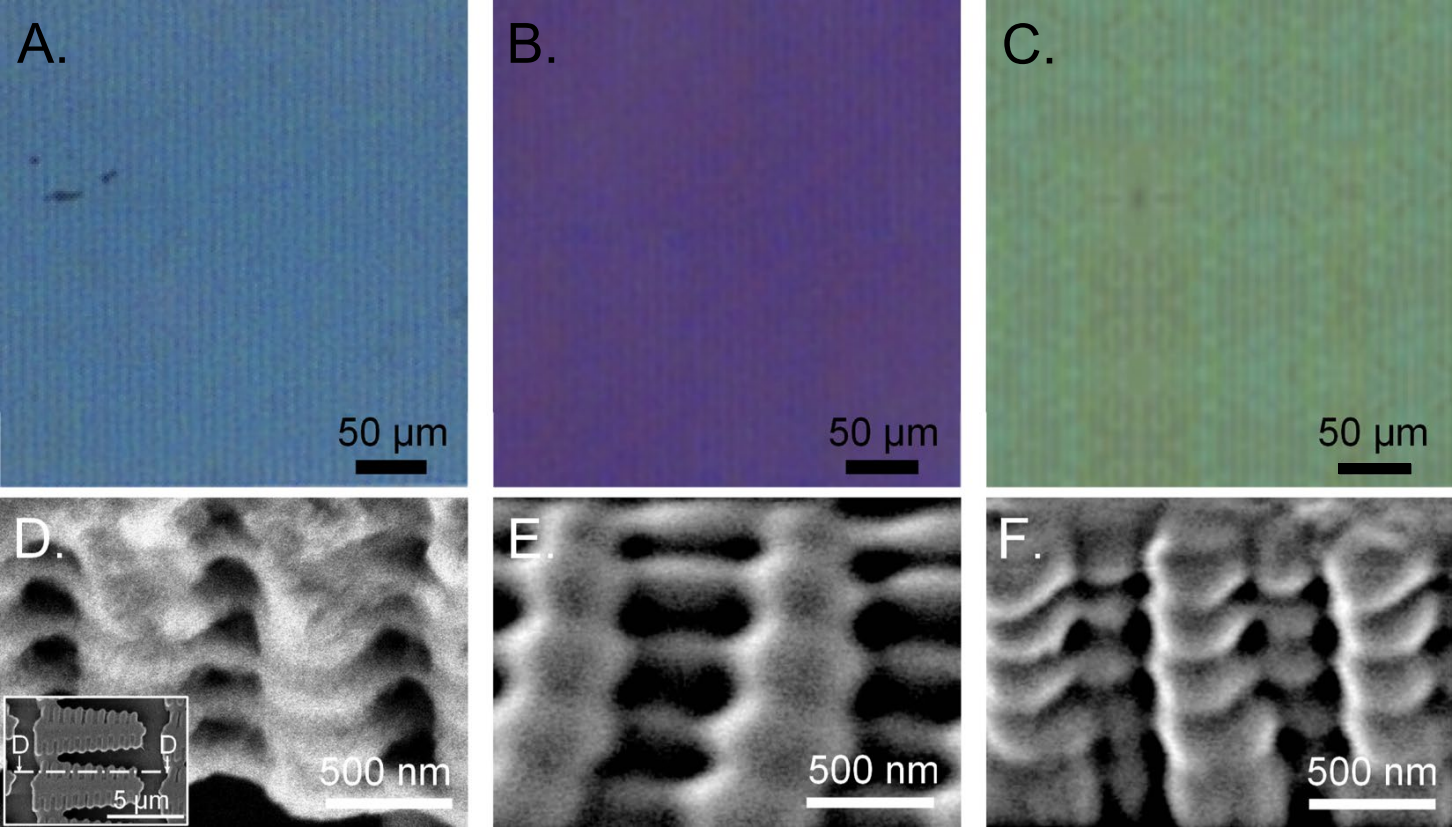
Mimicking the hierarchical surface micro-/nanostructures of the *Morpho* butterfly and coloration tuning by the variation of the phontonic structure’s size. The resulting coloration based on 2PP fabricated photonic structures are shown in (**A**)–(**C**). The associated SEM images of the microstructures are presented in (**D**)–(**F**). The oblique views of the microstructures demonstrates the hierarchical design containing air cavities and polymer lamellas. The total height of the microstructure was measured with white-light interferometry and is *L* = 1 μm. Since the number of periods is five, the total dimension of one air cavity and one artificial lamella for the resulting blue coloration (**C**) is 200 nm (*d*_1_ ~ 50 nm, *d*_2_ ~ 150 nm), which fits the air and cuticula dimensions inside the Christmas tree shape of a Morpho ridge. Whereas, the photonic microstrucutres of the purple coloration have decreased polymer lamellas *d*_1_ ~ 40 nm and increased air cavities *d*_2_ ~ 160 nm. The microstructures of the green coloration have the same amount regarding the dimensions of the polymer lamellas and air cavities (~100 nm).

To determine the existence of the microstructure hierarchical design, including the nanoscaled lamellas and interlayered air cavities, the samples were scratched and examined with an SEM. An uniform five-layer lamination consisting of air cavities and a thin polymer material was identified in all three samples and is shown for sample A in Fig. 6A and D. Since the total structure height is *L* = 1 μm (5 periods), the total thickness of the interlayer cavities and polymer lamellas is approximately 200 nm (*d*_1_ ~ 50 nm, *d*_2_ ~ 150 nm), which agrees with the total air and cuticula dimensions^8^ of the *Morpho* butterfly. The cross section of the lamellar 2PP structures was comparable to the shape of a Christmas tree (compare Fig. 1E) and mimicked the *Morpho’s* hierarchical ridges. The layer dimensions of the air cavities and artificial lamellas (see Fig. 5) are well comparable to those as in the case of the *Moprho* butterfly^8^. As a result of the similar design properties, the angle-independence of the coloration and a suitable polymer refractive index value, the artificial blue color formation has *Morpho*-like characteristics. Therefore, the color formation and its properties are based on a multilayer interference, diffraction and scattering.

Since the color formation depends mainly on the aforementioned interference condition for a multilayer stack, the coloration can be changed by varying the thickness of the polymer lamellas and air cavities^47^. The total height of the microstructure (*L* = 1 μm) and the number of periods were kept constant for all fabricated coloration areas. For a maximum reflection in the purple wavelength range, a periodic photonic structure with the characteristics of the *Morpho* butterfly was achieved for *d*_1_ ~ 40 nm and *d*_2_ ~ 160 nm with the usage of the same photosensitive material (see Fig. 6B,E). The thickness of the polymer lamellas and air cavities should be equal (~100 nm) to realize a green coloration (see Fig. 6C,F). The overall size for both features fits to the theoretical values calculated with the interference condition of a multilayer stack. The variation of those parameters was controlled by different laser power values. In conclusion, those results strongly support that the natural blue coloration of the *Morpho* largely depends on the effect of multilayer interference. The angle-independence of the artificial coloration can be explained by the imperfectness of the artificial hierarchical system.

## Discussion

Structural coloration with similar optical properties as the *Morpho* butterfly was clearly achieved in our experiments. Fully cured layers with the same thickness and cube-shaped polymerization only showed a high transparency. The primary microstructures written directly using 2PP (see Fig. 4) are also not responsible for the coloration because the dimensions are too large for wavelength interference in the visible range. Non-trivial light scattering was observed (see Fig. 5B) in the nanostructure presented in Fig. 6D. Although the primary microstructure (see Fig. 4) does not influence the structural color formation, its geometry is highly relevant. The nanostructures result from nanoscaled polymerizations between single branches via diffusion of radicals^45^ and they are spaced by an artificial ridge layer (see Fig. 2). The coloration of the produced samples can be detected from a wide range of observation angles (0°–60°) as illustrated in Fig. 5. The color’s intensity can be controlled via the density of the lamellar microstructures.

The generated coloration is non-iridescent. While the effect of iridescence depends on long-range order, e.g. multilayer systems^4^, non-iridescent structural colors in nature are produced by amorphous photonic biomaterials, i.e. short-range order^48^. Therefore, a disorder in the orientation of the polymer lamellas and air cavities (see. Fig. 6) inside a single periodic stack is responsible for the non-iridescence of the resulting coloration. Since iridescence is a great feature for organisms in nature to generate display functions^2,3^, this optical property is disadvantageously for the most real world applications, e.g. textile apparels or imaging devices. The amorphous property in our results depends on the deviation of the axial stage’s precision in our 2PP setup. Although high precision devices are obviously better suited for improving the 2PP process, the stage deviation contributes to an addition of disorder in the color system which can also be observed in nature. Therefore, the complex modeling of the exposure paths or the integration of complex algorithms to mimic disorder can be avoided. However, the degree of disorder in one of our artificial photonic structures is less in comparison to the disorder in natural color systems. All microstructures are highly ordered oriented in the fabricated area and the disorder can only be identified in the artificial periodic nanostructures. In contrast, the wing of the *Morpho* butterfly, for example, is more complex. The wing contains many hierarchically structured scales on different levels (see Fig. 1) which can be distinguished regarding the structures’ density and their orientation in 3d direction. The high degree of disorder leads to the optical properties of the *Morpho* butterfly^6^–^8^, and therefore, it leads to iridescence.

Our results demonstrate that bioinspired non-iridescent coloration can be generated by specific biomimetic disorder. The scattering of the total artificial color system did not correspond entirely to Rayleigh scattering or diffraction but to the specific color formation characteristics of the *Morpho* wing scales. The angle-independence of the artificial coloration can be explained by the imperfectness of the artificial hierarchical system.

Thus far, replicas of the Morpho lamellas can be artificially created using multilayer deposition^23,24^, FIB-CVD^25^, synthesis of nanostructures^26–29^, and e-beam lithography^30–32^. However, these fabrication processes require cumbersome tools and workflows that are neither suitable for research and development of a uniform physical concept to fabricate different structural color hues or for establishing a broad variety of recipes and rules to fabricate structural colors with different hues. For example, accurate replicas of the *Morpho* Christmas tree structure produced with FIB-CVD can only be fabricated on micrometer-sized areas^25^. The other methods are highly complex due to the usage of vacuum atmosphere, individual masks for the fabrication of different structure geometries^32^, different materials^23,24,31^, or multiple manufacturing process steps^26–30^.

We demonstrated for the first time in the present paper an effortless method for the generation of structural color that is much simpler than the aforementioned fabrication tools. This method uses 2PP to create an adapted cross-sectional geometry of the *Morpho* Christmas tree structure in a single photosensitive material. The results indicated that an accurate replication of the *Morpho* surface structure is not necessary, but only lamellar nanostructures that mimic the air and cuticula dimensions^8^ of the butterfly organism. The maskless fabrication of arbitrary structures using 2PP represents a research tool with considerable potential that for instance can be integrated in a computer-controlled closed-loop procedure for the geometrical design and characterization of the resulting structural colors to generate color hues over the entire visible wavelength range. The large angle-independence of the colors can be further increased by adapting the irregular distribution of the photonic structures on the wing of the *Morpho* butterfly^5^ to the 2PP manufacturing procedure. Furthermore, different colors can be simply generated by modifying the interference properties of the 2PP structures via the distance variations between the air cavities and the nanolamellas^47^. Thus, a purple and green coloration was fabricated artificially by different thickness values inside the microstructure’s periodicity (Fig. 6).

The results can be used to create new, innovative bionic products, such as optical components or technical color surfaces. For this purpose, the structural color geometry blueprints can be used in other lithographic methods to fabricate bio-compatible colors with a high throughput.

## Methods

### Sample preparation, development and analysis

Artificial structural coloration was generated in Femtobond 4B (Laserzentrum Hannover, Hannover, Germany). The photoresist is an organic-inorganic, biocompatible hybridmaterial with ceramic content and features the characteristics of transparency with a refractive index of 1.51 ± 0.02 for the visible spectrum of light. For the generation of a thin polymer film, Femtobond 4B was diluted with 1-propanol at the ratio of 1:3 and spin-coated with a rotation speed of 1000 rpm for 60 s on the glass substrate. Afterwards, the material had to rest for 24 h for the evaporation of the solvent. Photonic structures were developed in 1-propanol for 30 min after the 2PP fabrication process. Finally, the cover glass was cleaned for 1 min in acetone.

The thickness of the produced structures and the thin polymer film was measured with a whitelight interferometer (TMS-1200, Polytec, Waldbronn, Germany). The thin polymer film was cured with UV-light of a Hg-lamp (LH-M100CB-1, Nikon, Tokio, Japan).

### Fabrication of bioinspired nanostructures

A mode-locked femtosecond Ti:sapphire laser (Tsunami, Spectra Physics, Santa Clara, California, United States) with a repetition rate of 82 MHz, a pulse duration of 90 fs and a wavelength of 780 nm was used. The adjustment of the energy density of the laser focus was realized by a combination of a motorized λ/2-plate and a polarization beam splitter cube. The energy density was measured by a photodiode out of the average laser beam power. An acousto-optic modulator was employed as a fast shutter. The laser beam was tightly focused by an oil-immersed objective (100x Plan Apo, Nikon, Düsseldorf, Germany) with a NA of 1.4 trough a cover glass into the photosensitive material. The resist position along the axis could be controlled by mechanical stages (Wafer Max Z, Aerotech, Pittsburgh, Pennsylvania, United States). A CCD camera enabled the monitoring of the fabrication process. Arbitrary geometries were written by the deflection of the laser beam in the photoresist with the use of a 2D galvo scanner (hurryScan II, Scanlab, Puchheim, Germany). Furthermore, one mechanical stage (ANT 130-XY, Aerotech, Pittsburgh PA, United States) enabled the fabrication of an arbitrary number of structures on one single cover glass. In the experiments the scanning speed of the galvo scanner was 1.7 mm s^−1^. The blue coloration was fabricated with an average power of the laser beam of 14 mW. The purple coloration (11 mW) and the green coloration (18 mW) were generated with a different adjustment of the average laser power. In the process, Femtobond 4B was cured alternating layer by layer starting from the bottom of the coverglas. The distance between each layer was defined with 200 nm.

### Color monitoring

A microscope (Eclipse LV 100, Nikon, Düsseldorf, Germany) with a 12 V-50 W halogen lamp was used to study the color formation with brightfield. The monitoring occurred through a CCD camera (DS-Fi2, Nikon, Düsseldorf, Germany) using a microscope objective with 5x magnification. The halogen lamp illuminated the color area homogeneously in all directions. All filters were removed. The brightness of the halogen lamp was kept constantly for all results. To study color formation as a function of the viewing angle, an adjustable ramp was used to tilt the sample.

### Spectral Measurements and simulation

Samples were illuminated at 45° with a light source (DH-2000-BAL, Ocean Optics Inc, Dunedin, Florida, USA) through an optical fibre (200 µm diameter) with an one lens condenser mounted on it. The condenser was placed 10 mm away from the sample. Reflected light was collected by other condenser mounted on an optical fibre (200 µm diameter), which was connected to the monochromator (Ocean Optics Inc, Dunedin, Florida, USA). The spectra were recorded with the software Spectral Suite (Ocean Optics Inc, Dunedin, Florida, USA). Further spectra processing includes the dark noise subtraction, normalization of the spectra on the illumination source spectrum, smoothing with Savitzky-Golay^49^, and normalization on the area of the spectra. The reflection spectrum for multilayer systems was calculated using OpenFilters (Boston, USA)^50^. For the spectrum calculations the multilayer system on 0.14 mm thick borosilicate cover glass contained five periodic layers of 2PP polymerized Femtobond 4B and voids with a total thickness of 1 µm. Unpolarized light was incident at 45°.

### Scanning electron microscopy

For SEM imaging the samples were mounted on aluminum stubs by using double-sided carbon conductive tape (Plano, Wetzlar, Germany) and sputter coated in a BAL-TEC SCD 500 Sputter Coater using a BALTEC QSG 100 Quartz Film Thickness Monitor (Bal-tec AG, Balzers, Lichtenstein) with 10 nm thick gold-palladium. After preparation the samples were investigated using a scanning electron microscope Hitachi S-4800 (Hitachi High-Technologies Corp., Tokyo, Japan) at an 3 kV accelerating voltage.
